# Supplementary figures and images for: Health impacts of industrial mining on surrounding communities: Local perspectives from three sub-Saharan African countries
Source: PLoS One. 2021 Jun 4;16(6):e0252433. doi: 10.1371/journal.pone.0252433 (PMC8177516; doi:10.1371/journal.pone.0252433)

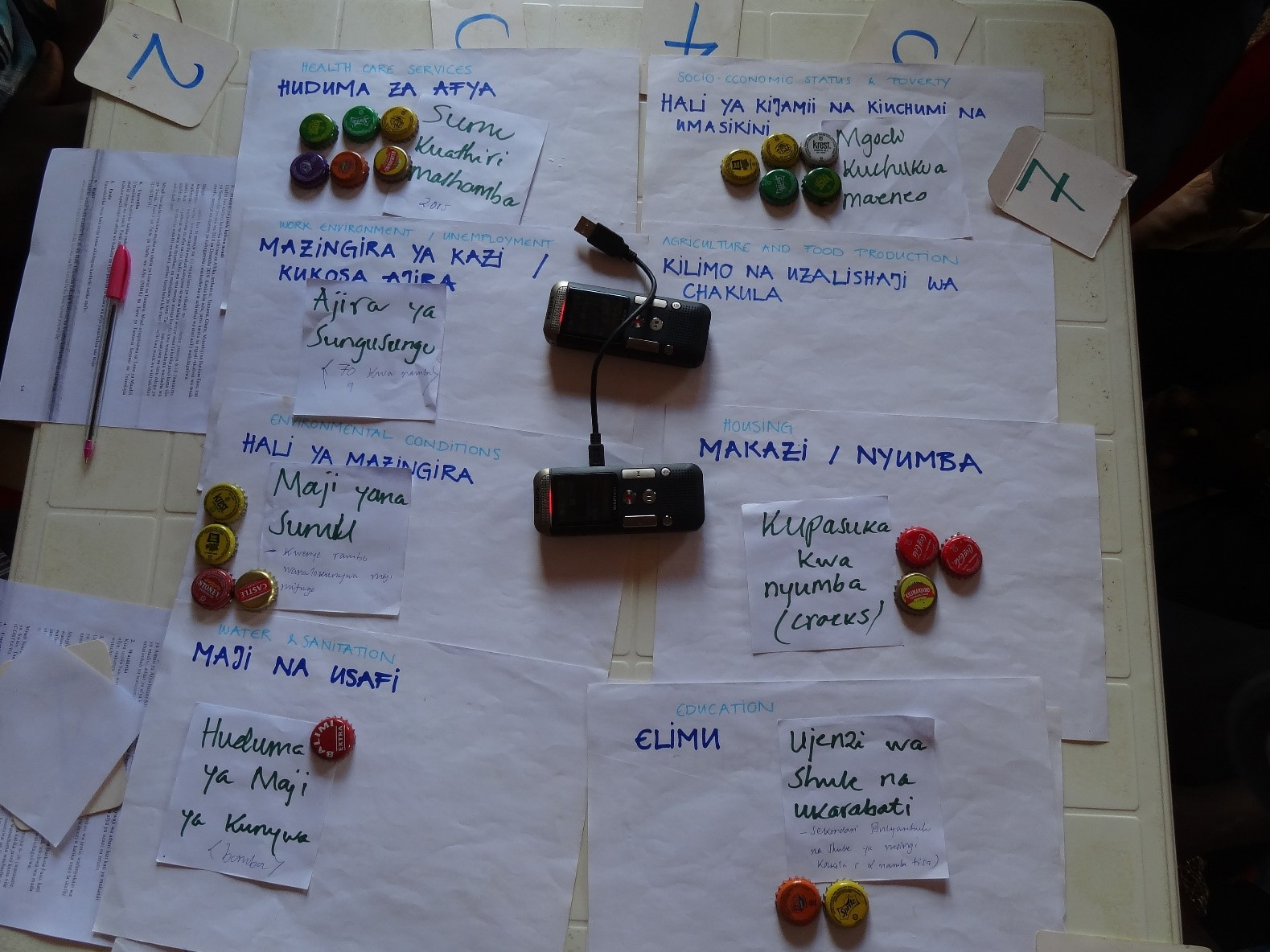

Supplement: S1 Fig — The picture shows the perceived impacts (written in Swahili on paper cards), which were categorised by the participants on the wider determinants of health (A4 sheets) and ultimately ranked with bottle lids. (TIF) [file pone.0252433.s001.tif]
