# Supplementary material for: Health impacts of industrial mining on surrounding communities: Local perspectives from three sub-Saharan African countries
Source: PLoS One. 2021 Jun 4;16(6):e0252433. doi: 10.1371/journal.pone.0252433 (PMC8177516; doi:10.1371/journal.pone.0252433)
Supplement: S1 Table — Thematic codes for the perceived impacts on the wider determinants of health, description of the themes and exemplary quotes extracted from the transcripts of the FGDs. (PDF) [file pone.0252433.s002.pdf]

S1: Excerpt from the codebook

Thematic codes for the perceived impacts on the wider determinants of health, description of the themes and exemplary quotes extracted from the transcripts of the FGDs.

Name	Description	Exemplary quotations*
**Perceived enviornmental impacts**		
Environmental	Statements related to environmental conditions in general such as geographical situation, environmental degradation or climate conditions	- Les dégâts environnementaux seront énorme<br>- As condições climatéricas desta comunidade costuma estar ao contrário<br>- As you can see near that pit there is a mountain that when it rains the mud from the mountain can collapse
Air pollution	Statements related to air pollution or dust in general or not specified (with one of the child nodes)	- La poussière nous dérange beaucoup<br>- Defecamos fezes com cor preta por causa de poeira<br>- Air that we breathe is not clean
Bad smell	Statements about odour or bad smell (around the mine or along a mining pipe)	- L'odeur empeste tout le village<br>- Sai um cheiro mau, e nos faz sentir mal aquele cheiro<br>- The smell of chemicals here are strong<br>- This bad smell is emitted by a pipe, it goes straight on air
Blasting - dust from inside the mine	Statements about dust coming from inside the mine, dust from the mining activities	- Quand ils écrasent leur or, il y a la poussière qui se repend dans le village et on ne peut plus regarder<br>- Consegue ver as poeiras saindo das minas para dentro da vila. O ar que temos respirado, é poluído<br>- The blasting they do here releases dust into the atmosphere<br>- The blasting pollutes the air that we breathe<br>- There is a dust coming from inside the mining
Traffic - dust	Statement about dust related to traffic on unpaved roads	- Esses carros deles quando estiverem a passar costumam nos sujar com poeira também<br>- I want to add something about dusts, mining company is using big trucks when passing to our community dust comes to our houses
Water	Statements related to water in general, i.e. when participants were speaking simply	- On demande qu'ils nous aident avec de l'eau<br>- Água conseguimos mas o governo e a Kenmare não nos abastece água<br>- Water is life and our health depends on water, if water is not safe then our health is also not safe.

		about "water" without specifying the source or issue related to it	- We get diseases like chest pain, stomach pain and other diseases and the main cause of these diseases is the water we are using
	Burst of mining dam - water flow from the mine	Statements related to the burst of the tailing dam (in the mine) or when (contaminated) water was flowing from the tailing dam to the communities.	- En saison pluvieuse il y'a des coulée qui contamine les eaux de surface que les animaux boivent<br>- Sai água dali [da empresa] que vai aqui na comunidade e essa água misturasse com a água que nós bebemos<br>- Last year the dam was full of water then it broke, water flowed to rice plants which dried because of the chemicals.<br>- They are constructing dams but when the dams are full water flows outside then poison which is in the water flows to another dam
	Ponds as breeding sites	Ponds or surface water related to vector breeding	- Lugares que tem charcos ou mesmo dentro de casa quando tiver água na bilha que leva muito tempo dentro de casa isso provoca mosquitos acaba picando as crianças mesmos adultos<br>- Here are ponds where mosquito breeds and spread malaria<br>- The stagnant water behind our homes to breed mosquitoes
	Type of water source	To specify type of water source with the given sub-nodes or to code segments were multiple water sources are mentioned	
	Dams - ponds - rivers	Natural water bodies, surface water	- L'eau du barrage de la mine affecte nos retenues d'eau en brousse<br>- A água que cartamos no rio Zambeze apenas bebemos, a água não é tratada<br>- On the dam which was constructed for animals to get drinking water but water has poisons<br>- When it rains water from the mining flows to the river then to our village
	Piped - drilled water points - taps	Water infrastructure for water supply	- Certaines personnes partent jusqu'à Sabcé acheter l'eau à la borne fontaine<br>- Nous avons eu à réparer notre seule pompe du village<br>- Montaram aquela bomba para poder nos ajudar quando lá no rio a água ficar suja<br>- We should request to put pipes to distribute water to this community<br>- they brought water services and all these water taps
	Rain water	Rain water, rain water collection systems	- Cette eau des toits est devenue gluante<br>- Avant nous recueillons les eaux de pluie qui coulaient des toits de nos maison<br>- Pode a chuva cair não vem boa água<br>- Quando estiver a chover caí água suja de carvão na chapa<br>- We cannot even dare to fetch rainy water for drinking

Wells	Natural wells, unprotected wells, open wells	- [La mine] est entrain de contaminer notre eau des forages<br>- Nous nous inquiétons de la potabilité de ces eaux de puits<br>- Costuma estar difícil, porque todos nos cartamos água no pequeno poço<br>- We are using open natural wells
Water access	Statements related to physically being able to access water or when they are referring to the distance of the water source, also when they cannot access water for free	- La distance lointaine de l'eau rend certaines choses difficiles<br>- A água estamos a pagar muito carro<br>- Essa água não estão nos a dar, estamos a pagar<br>- Agua está distante<br>- It is located far away from the community, so people are choosing to fetch water from the nearby well instead of going there
Water as habitat	Water as habitat for any kind of animals	- Quando vamos cartar a água não enche uma lata e está cheio de areia, abelhas e sapos<br>- Quando vamos no rio, somos pegues com crocodilo<br>- Water had poison because leaders from the mining visited us and I was there, fish and other living organism died
Water availability	Statements about the availability of water and water access points	- L'eau ne suffit pas<br>- On veut aussi qu'il ait beaucoup de pompes pour qu'on gagne assez d'eau<br>- Não temos água<br>- We also have shortage of clean water<br>- They constructed wells for us
Water quality	Descriptions about the water quality (colour, taste or smell, appearance/composition) as well as the measurement of the water quality	- L'eau est trouble, la couleur de l'eau change<br>- La mine fait des prélèvements d'eau à des fins d'analyse<br>- Nós a bebermos água suja<br>- Depois essa água está contaminada<br>- There is a dangerous poison in water
Sanitation	General statements about sanitation and sanitary infrastructure such as toilets	- La majorité des latrines de la cité sont pleines<br>- Aqui na comunidade não habituamos latrinas, estamos habituados a ir fazer (cocô) ai no mato mesmo<br>- We have worked so hard to push them to construct sewage system
Hygiene	Personal hygiene (bathing, showering, washing hands) and household hygiene (food hygiene, cleaning the house, washing clothes)	- On dit qu'il y a la santé parce que si tu es une femme et que tu assainis ton milieu<br>- Tomamos banho uma vez por dia<br>- You can see we are living closer to this road and there is dust everywhere even on the food

Open defecation	Descriptions of defecation and urination of employees of the mine or community community members	- Il[s] [les agents de la mine] défèque[nt] dans les champs<br>- Outras vezes encontra enquanto cagaram (refere-se a defecar) lá na sua machamba<br>- Security guards working outside the mining are making our environment dirty because they don't have toilets
Waste management	Waste management of the community and the mine	- Parfois jette les restes de repas pourri dans nos [champs]<br>- Temos uma lixeira que não esta protegida, só o lixo e deitado (o lixo é despejado ao relento)<br>- Those chemicals are dangerous to health of human being if are not handled properly, this means people living in the community surrounding the mining can be affected too<br>- There is no damping place to dispose rubbish
Soil and land	Heading category for issues related to soil and land (general statement or when it is not clearly linked to a child-node in the subcategory)	
Agriculture	General statements related to agriculture and the productivity of agriculture (increase or decrease), therefore when the reason for the change in productivity is not clearly stated and the quotation cannot be coded by a following child-node. Agriculture considered as subsistence work or income generating activity.	- De nos jours c'est très difficile de cultiver ici et pas de rendement.<br>- A Mineradora estragou as nossas terras, porque antes cultivávamos bem<br>- Agriculture production is very poor
Destruction of fields	Physical destruction of agricultural fields by machines or other activities of the mine	- Porque encontrão sua machamba eles pisam, estragam seus produtos, isso tudo provoca-nos fome, a senha para receber sementes não dão
Food	Agriculture as food production; cultivating and harvesting crops and vegetables; quantitiy and quality of food (from the fields)	- Les champs qu'on a demandé ce sont de petits champs et quand on récolte ça nous suffira pas jusqu'à la saison prochaine<br>- *Carakata* (farinha de mandioca) está toda estragada<br>- It doesn't matter how hard you will try to cultivate but you won't get enough food<br>- Dusts affect us so much because of eating poison
Land taken	Loss of agricultural land or fields	- C'est le blanc là [from the mine] qui a fait qu'on n'a plus d'argent parce qu'il a pris nos terres où on se débrouillait.<br>- As machambas levaram com a empresa; levaram sim com a empresa e não nos deram nada<br>- To be honest by taking our land they have killed us

	Less rainfall	Reduced production (agricultural or fishing) due to less rainfall	- We were getting a lot of crops but since they started doing mining activities rate of crops has dropped because we don't get enough amount of rainfall<br>- Não há chuvas, até peixe já não temos
	Resettled	Size of land or soil fertility in the new area, distance of resettled fields	- C'est parce que les champs sont très éloignés de nous maintenant<br>- A empresa [...], indemnizou a todos que eles atingiram para que pudessem abandonar as suas machambas e procurou machambas para a população afetada.<br>- When we moved out we left our fertile farms and productive then we moved to new area where the farms are not fertile.<br>- They shift me and take me to another place where there is no manure, so everything that I cultivate in a new farm doesn't give me a good outcome
	Soil pollution - drought	Drought or polluted soil (through air, water or soil) and related consequences for the growing plants	- Amendoim é amargam muito, quando plantas não cresce porque ai a terra já não esta boa<br>- Poison water flowed to rice farms and plants were burnt and since then plants are not growing in those farms because the land is affected.<br>- We are not safe because when it rains water comes from the dam to our crops, when that happens our crops get dry
De-forestration		Cutting down trees or forest, clearing of woods and loss of herbs (inside the woods) and firewood as well as related interventions (replanting trees)	- La mine a détruit tous les arbres qui existaient<br>- Eles [the company] até vieram distribuir plantas numa das reuniões que tiveram com a população. Eles deram plantas que não tem interesse, não são plantas frutíferas.<br>- Ai na mata eles estragam tudo, partem as árvores e deixam ai mesmo de qualquer maneira<br>- Because clearing of the forest has let to shortage of rainfall in our country<br>- We had certain trees that we used for traditional medicines but we don't get those trees anymore
Land accessibility		Physically being able (or not) to access the land, physical barriers	- Parce qu'ils ont clôturé les lieux avec le grillage<br>- Nem pisamos na mata, custumam nos proibir, até colocaram guardas com armas só para nós não entrar, fizeram uma vala grande para nós não conseguir atravessar, porque quem tenta atravessar a vala morre logo
Pastoralism - animals		Statement related to domestic and wild animals, domestic and income-generating activities with animals, ONE health aspects (interaction of animals and humans which are relevant for their health)	- Ils avaient promis nous donner des animaux à élever<br>- L'élevage à aussi beaucoup de pans: il y'a l'élevage des montons; des poulets; des bœufs<br>- Até mesmo animais, há dias atrás morreram por ai 4 cabeças de Gado Bovino, estava doente. Quando morreu abrimos a barriga e vimos que toda a barriga estava escura, devido a esta água<br>- Animals are drinking water which has poison and eat grasses which have poison

Housing	Heading category about housing and living conditions, compromises general statements, which cannot be specified by the following child-nodes (e.g. statement related to interventions on the housing communities or when reason for improvement or impediment are not specified)	- On y trouve maintenant de belles constructions de maisons<br>- They gave houses to people but the houses have even broken down, they were not of good quality
Blasting - vibration - cracks	Statements related to the effects of blasting on the houses or living area or cracks in the houses	- Depuis que la mine est installée, on constate les fissures des maisons<br>- Rachas nas casas. Quando eles explodem Os dinamites, primeiro a terra treme depois as chapas de casa se levantam, quando estiver a chover caí água suja de carvão na chapa.<br>- Blasting comes with vibration which causes cracks on our houses<br>- At that time there was blasting at the mining, there was very strong land vibration.
Electricity	Statements related electricity	- Costumo colocar painel solar para poder carregar meu telefone<br>- The electricity passes to our farms and inter in the mining but we don’t have electricity. So we would like to ask that if there is a possibility, they should bring us electricity<br>- At night this community is in darkness, no electricity
Land - resettlement	Owning land and land rights, resettlement of home or land for living (compared to land for agriculture), construction of new houses	- C’est la société minière qui nous a dit de choisir un lieu dans nos domaines afin qu’elle construise les maisons pour nous<br>- Parce que quand tu n’a pas les documents d’une parcelle, alors tu es en insécurité foncière<br>- Sans ces documents, nous ne sommes pas propriétaire de ces maison<br>- O importante, é que sejamos reassentados num outro local que não tenha mineradoras<br>- The pit they dug was right in front of our house so they relocated us to a new house<br>- We don’t know whether we live near the mining or we live within the mining area because their beacons are here<br>- By law we are not supposed to be here
Noise – noise pollution	Acoustic effects of blasting or machines used in the mines, potentially also traffic noise	- Quand tu es couché au petit matin tu as peur du bruit<br>- Tu perds par moment le sommeil à cause du bruit «kououououoooo» des engins lourds<br>- Não apanho sono, quando explodem até fico assustada<br>- We don’t get used to the blasting sound people are shocked whenever they do blasting
Roads	Statements related to the road network in the communities or the quality of the road and implication for the mobility of the communities	- Ils nous ont promis construire une grande route<br>- As estrada estão todas esburacadas desde que chegaram e começaram a trabalhar ainda não arranjaram<br>- They came to level[ed] our roads

		- Construction of this road to town has made movements to the community and outside the community to be easy.
Traffic - trucks - accidents	Statements about the traffic in the community and related consequences for the safety of the communities	- Quand ils passent avec leur véhicule ils tuent (cognent) nos animaux (les poules et les chèvres meurent) de plus la poussière nous donne des maladies.<br>- Lá na cidade sempre tem havido acidentes, quase todos dias, principalmente, nos finais de semana<br>- Heavy trucks passing on the road which causes vibration which affects our houses<br>- This road is used frequently there are so many tracks taking things to the mining and out of the mining.
**Perceived impact on social services and organization**		
Social	Heading category for issues related to social services (such as education and health care services) and social organisation of the community	
Education - schooling	General statements related to education or when not specified for one of the given child-nodes (not inlcuding higher education or educational programmes)	- Escola construíram, construíram escola industrial só as pessoas daqui é que não estudam<br>- Education is the source of better life for our children in the future
Attendance - accessibility	Being physically able to go to school and attend the classes (or not attend)	- La mine a fait installer un grillage, lequel grillage a augmenté la distance entre koussaro et le collège puisqu'elle barre l'ancienne voie<br>- Temos escola industrial, só não frequentamos, lá tem coisas, as crianças não vão só fizeram mas não vão.<br>- Now our school can manage to take many students<br>- Children will be stressed they cannot even go to school because they are thinking of how can he/she help his old father
Buildings - infrastructure	School buildings and infrastructure in and around the classrooms	- Ils ont construit des salles de classes<br>- Construíram duas escolas que leciona até Oitava classe (8ª Classe) aqui na CFM e me disseram que a [empresa] que construíram?<br>- They have supported construction of two secondary schools<br>- They brought iron sheets, wood and color but they didn't gave money to build the school instead they gave those things to village government.<br>- They built classrooms here [in the village], also teachers' houses and toilets

School fees	Financial aspects related to education, being able to pay or get support for paying school fees	- Aucun des parents n'a de l'argent pour payer la scolarité<br>- Tinham dito para entramos na escola e estudar, mas na escola nos pedem dinheiro<br>- They are giving support to orphans children in this community to get education<br>- They confirm that there are some children who are poor and they are being sponsored by the mining in their education
Health care services	General statements related to health care or health care services or when not specified for the given child-nodes	- Os médicos vão dizer que não acusou nada nos testes e análises feitos no hospital<br>- There is reduction of maternal and infant deaths in the community<br>- There is no improvement on health services
Accessibility	Statement related to physically being able to access health care or distance that they have to travel to access health care	- Le centre de santé dans lequel nous nous rendons en cas de maladie est éloigné de notre zone<br>- Se tivéssemos um hospital perto estaria a nos ajudar a tratar das nossas doenças e as pessoas não estariam a morrer descontroladamente.<br>- Now it is easy to go for delivery for us women<br>- So when mining built this health center here we feel good, we get treatment near our homes
Affordability	Financial aspect of accessing health care services including transport costs to get there	- Si tu es malade pour te soigner il faut de l'argent pour les soins sans argent la maladie va s'aggravé jusqu'à ce que tu vas trépasser<br>- Nós pagamos ambulância para levarem nosso doente ao hospital em Maputo para ser operada<br>- Because people financial situation is poor, they cannot afford to pay for the health services even when someone is in need of it<br>- We don't waste transport fee as we used to do before
Availability	Availability of health care facilities, for example when new health facilities were constructed	- Ils ont construit un dispensaire<br>- Lamentamos por causa de não ter hospital?<br>- We don't have even a dispensary in this community
Capacity	Capability to receive or treat people (number of beds or waiting time)	- Apenas é um hospital somente e as pessoas são muitas?<br>- The waiting time to get services is shorter […] you don't spend the whole day waiting to get treatment<br>- The [XX] health centre has helped in decreasing the crowd of people at [XX] district hospital
Readiness	preparedness of the health facilities for treatment, availbe equipment or availability of tests and medicines,	- La mine avait appuyé le CSPS en médicaments pour les enfants<br>- Disseram que não temos outros comprimidos a não ser aspirina?<br>- You can have a building of dispensary but it is not full equipped for it to provide services to community members<br>- After the mining renovated health centres, hospital buildings, providing medical tools and other things

Seeking health care	Frequency of seeking health care, description of reasons for seeking health care and places where they are seeking care (hospitals, traditional healers, pharmacies)	- Les gens achètent un comprimé appelé F sur la place du marché qui les soulagent<br>- Quando podemos temos ido ao hospital, mas não está a resultar, é preciso que tenhamos o hospital aqui perto<br>- Now majority are going at the health facility to do checkup before using medicines while in the past people went straight pharmacy to buy medicines<br>- Currently majority are going hospital when they are sick not to traditional healers as it was in the past
Specific services	specific health care services provided, treatments offered or health care infrastructure constructed	- Ils nous ont promis une ambulance<br>- Quando vão fazer parto no hospital, e se ser difícil o parto muitas mulheres levam cesariana e voltam com duas crianças mas isso há muito tempo não acontecia<br>- They have educated people and there is testing of HIV frequently in our community<br>- They have provided two important services, children with open-cleft and children who had fire scars they took them somewhere and treat them<br>- Also they built a mortuary
Community dynamics	General statements about the community dynamics or when not able to specify with one of the following child nodes	
Community conflicts - inequities	Conflicts and tensions among community members or subgroups, when not everyone is benefitting equally and certain people are privileged compared to others	- L'arrivée de la mine a été bénéfique pour certains et une perte pour d'autre<br>- Nossos dirigentes tem muita "doença" (problemas ou desentenimentos) com a comunidade de [XX], não querem nos ver a trabalhar<br>- The mining paid the money with agreement but the leaders did not educate the community or they were forcing people to sell their farms<br>- Few opportunities are offered to the point it brings conflicts to community members.<br>- She has a referee. (*She knows someone in the mining who has connected them to get employment opportunity at the mining*)
Crimes	Committed crimes like robberies, stealing and thefts within the community or in the mine, also crimes related to ongoing conflicts	- Si les voleurs n'avaient pas volé nos animaux, ont pouvais vendre quelques animaux pour acheter les condiments<br>- Não estamos bem, temos medo por causa de assassinatos; não sabemos de onde vem essas coisas, estamos ouvir *Al Shabaab*<br>- They are stealing our properties in the community<br>- When the mining started operating they remain jobless and that is why they started stealing in the mining
Culture - beliefs - future	Statement related to the culture (like rituals), beliefs (like myths or religion) or	- Ils sont venus nous dire de faire l'élevage des porcs nous on a dit que nous sommes des musulmans et que nous ne pouvons pas le faire

		anticipation of effects in the future (for the future generation)	- La présence de la mine a chassé les génies et les démons de la brousse et ils ont habité les gens maintenant<br>- As pessoas pensão que agora tem má sorte, eles abrem furos e sai azar dai mesmo<br>- We are living by God's grace and protection<br>- So what will support my children in the future?
	Direct contact contamination	Transmission or spread of diseases through physical or sexual contact of people	- Ficam cheio de doenças e as nossas filhas dormem com esses Sul-africanos, esses de Maputo; está a ver, são dominadas assim apanham SIDA amanha morrem<br>- Então essas nossas filhas que dormem com esses Sul-africanos dizem que são dois, mas esses dois são muitos, nossas filhas morrem, as mães morrem
	Family - cohesion	Family or community spirit of feeling connected or belonging together, as well as trusting and respecting each other, respectively when this is all not given anymore	- Les femmes se frappent souvent au niveau des pompes<br>- Crianças não tem medo, encontram um adulto passam mas antigamente as crianças se ajoelhavam *ruwa*, *ruwa* ali diziam que filho de fulano sim tem respeito<br>- Há muito tempo era normal você, a andar no bairro, encher um saco plástico de amendoim de oferta; as pessoas oferecerem-te só, mas agora isso não existe; ninguém dá o outro, se você não tem estás mal<br>- When they take the money they run away from their families<br>- They get involved in adultery and end up getting infection
	In-migration	Migration to the communities, increase in population, mine as pull-factor	- La population de la cité à fait accroitre la population qui existait de [XX]<br>- E essa vinda de pessoas de outras regiões, faz com que surjam novas doenças na comunidade<br>- Many people are coming here looking for employment opportunities in the large mining
	Security	Feelings about safety and security (positive and negative)	- Partout où tu passes en brousse on te chasse<br>- They were also announcing on radio that no farmer should use their lands not even for the slightest activity otherwise when a mining soldier sees you, he/she can arrest you<br>- After the phase out of mining activities the pits will be very dangerous to the living things that is human being and animals<br>- This has cause the area to like a forest and people are raped and sometimes killed because the area has been left idle<br>- As villagers we are benefiting due to the security services provided by these youth, they are our guards so in one way or another we are benefiting from them
	Sports teams - interests groups	Formation of or (financial) support for local groups or associations (e.g. sports team, beekeepers, vegetable gardeners)	- On a créé aussi un groupe de femmes qui creuse le zaï<br>- Eu quero pedir a [empresa] temos desporto em particular futebol<br>- They sponsor football teams<br>- They tried to educate people on the modern agriculture, we have groups where we are being educated on how to perform modem agriculture

		- We have introduced women groups which basically support each other financially
**Perceived economic impacts**		
Economic	Heading category for issues related to economic aspects or general statements about economics that cannot be specified with one of the following child nodes	- Our economy here might be a lie
Compensation	Compensation payment for resettlement, relocation of land or house as well as indications about how this money was spent	- Ils ont donné de l'argent à ceux qu'ils ont pris les terres.<br>- Quand on déménageait dans la cité, dans chaque concession ils ont donné la somme de 200000F CFA<br>- Mas a empresa já nos dominou nos arrancou nossas machambas os que tiveram a sorte de serem indemnizados uma machamba grande cheia de produtos só lhe davam 10 a 20 milho meticais<br>- Native who got paid had a lot of money, they used the money for drinking, seducing students and marry other women<br>- People were paid and shifted then they used money badly
Economic benefits	other economic benefits (not related to income generating activity)	- Dizem que cumpriram dar pensão aos idosos<br>- Omem outras pessoas (presumivelmente quis dizer que "outras pessoas é que tem o beneficio da riqueza que a região possui") [...] esta é a comunidade que tem recursos minerais e não acreditar porquê do jeito como está não espelha a realidade<br>- That 10,000 from security guard is for street [community] development<br>- I didn't use my money to contribute on the building of classrooms and teacher's houses, they helped us on that
Income generating activities	Means of livelihoods, subsistence work or businesses driven by community members (compared to employment by the mine), such as artisanal mining, farming, fishing, prostiution	- Le marché ne donne plus, tu vas cultiver mais pour vendre, tu ne trouves pas le marché<br>- On faisait l'orpaillage et on avait toujours l'argent<br>- Demain chez vais préparer mon dolo (bière de mil) pour vendre et avoir de l'argent<br>- As pessoas saiam de outros pontos para virem comprar mandioca fresca aqui mas nesses dias não<br>- Ficamos toda hora no rio ir pescar peixe porque há falta de emprego.<br>- People are selling their bodies (*prostitution*) to satisfy their needs<br>- Youth are going steal some abandoned stones in the mining so that they can come and process it and get small amount of money for their needs
Living costs	Expenses for living and domestic activities, sometimes specified goods that they have to encur now, as they are not	- Acheter l'eau à la borne fontaine<br>- Nous ne disposons pas de moyens pour les réparer<br>- On avait les amandes de karité que nous ramassions pour faire du savon et depuis que nous sommes ici, nous n'avons rien et si tu as besoin du savon il faut en acheter

	available anymore naturally because of the mine	- A realidade do povo que não conseguem comer arroz porque custa 2 milhão e 500mt<br>- When you get money you go and buy food, you won't buy manure<br>- Where will you get money for medication?<br>- Life running costs increased<br>- We don't get firewoods so we are enforced to buy charcoal
Poverty	Poor economic status, poverty	- La paupérisation de la population<br>- Só sabem trazer pobreza e fome para as nossas comunidades e não temos o que comer<br>- People financial situation is poor
Unemployment	Not being employed by the mine or not having work at all	- Pas d'embauche à la mine<br>- Está tudo estragado e não apanhamos emprego, e nossos familiares não apanham emprego também<br>- Aqui não há emprego para nós, quem apanha emprego são pessoas que vem de [the city], são as pessoas que conseguem trabalho na fábrica<br>- We are starving out of unemployment<br>- Due to lack of employment our children turn to robbers
Working conditions and job opportunities (at the mine)	Job opportunities offered by the mine (employment conditions) and working conditions when employed by the mine such as salary, type of contract or occupational health issues	- Il y a aussi certains qui mangent la bonne nourriture à la mine<br>- Les gens arrivent à entretenir leurs enfants par rapport à avant, car ils y'a des parents qui travaillent à la mine<br>- Pode passar muito tempo, eles não lhe ajudam com a sua doença e se você está doente e trabalha lá e se você não for ao trabalho, logo acabas por perder emprego<br>- They are not giving natives long term employment although we are grateful natives have been given security guard posts<br>- The mining provided employment through the government and the government employed security guards and that is where we get that money that we are discussing<br>- Most of mining employees get sick due to the dust in the mining,<br>- We are short term employees at [the mine]<br>- Employment opportunities which are offered to natives is *sungusungu* (*local security guards*), it is kind of employment which we are not happy with because it is the lowest posts at the mining

* Participants speaking in the third person plural (they, their, them), they usually refer to the mine; before and after refers to before or after the implementation of the mine. Square brackets ([...]) are used to indicate changes made from the original transcript (e.g. to censor proper names or places).
