# Supplementary material for: Health impacts of industrial mining on surrounding communities: Local perspectives from three sub-Saharan African countries
Source: PLoS One. 2021 Jun 4;16(6):e0252433. doi: 10.1371/journal.pone.0252433 (PMC8177516; doi:10.1371/journal.pone.0252433)
Supplement: S2 Table — Complementary table to Table 3 with statements in original languages of selected quotations including of positive and negative health outcomes, categorized by morbidity, mortality, well-being as of the coding system. (PDF) [file pone.0252433.s003.pdf]

Original quotations of coded health outcomes

Complementary table to Table 3 with statements in original languages of selected quotations including of positive and negative health outcomes, categorized by morbidity, mortality, well-being as of the coding system.

	Positive	Negative
**Morbidity**		
Diseases	- On dépose le savon au bord des toilettes pour qu'après les selles on se lave les mains **afin d'éviter les maladies** (BF2_L2)<br>- Agradecemos essa rede que trazem porque reduz **um pouco a picada do mosquito** (MZ2.2_L1)<br>- Agradecemos porque vamos ao hospital porque […] vamos para **receber medicamentos** e esses da [empresa] dão e tomamos (MZ2.2_L7)<br>- Now due to the presence of a dispensary children are taken there for testing and if they **diagnosed** with malaria they are given medicines (TZ3_L2)<br>- There is **testing** of HIV frequently in our community (TZ2_L3)	- C'est depuis l'implantation de la mine que **nous tombons malade** (BF2_L7)<br>- A empresa provoca **muitas doenças (**MZ2.2_L1)<br>- We were using unprotected wells, they had no much effect but after the mining [...], now there are **many diseases** and we think it is caused by water (TZ2_L7)<br>- Dust is produced when they are grinding stones and this dust is spreading to the community where people are living. As a result people are getting **cough** (TZ2_L3)<br>- Also there is an increase of HIV **transmission** because many people from different regions came to work in the mining (TZ2_L1)
Accidents and injuries	*n/a*	- Les **accidents** de circulation qu'on y rencontre de la part des véhicules et de leurs camions (BF1_L4)<br>- [Um carro] da empresa Kenmare **quase que me atropelava** (MZ2.1_L1)<br>- If adult caught for stealing Magwangala [ore in the mine] are **beaten** so badly because you are matured enough to know what you are doing is wrong (TZ3_L7)
Disabilities	- We have good cooperation with the NREP [natural resource extraction project] we are allowed to employ this woman who is a **cripple (**TZ1_L6)<br>- The mining sponsors for medical treatment for children who have midomo ya sungura [**open-cleft**] (TZ3_L3)	- Ils sont venus tout fouiller avec leur machines font que ces génies étaient tranquille commence à ce mélanger au humains ce qui fait que certaines femmes mettent **des enfants bizarre** au monde (BF1_L3)<br>- Essas doenças é que estão mais cheias agora diferente de antigamente aqui, esse assunto de **Romatismo** é criando por causa da poera (MZ2.1_L2)<br>- I was working in the mining but later they discovered that I have **sight problem** (TZ1_L3)

**Mortality**		
	- Nós agradecemos essa rede que nós dão […] Se não fosse **rede** ninguém ia sobreviver aqui na comunidade (MZ2.2_L11)<br>- We were travelling long distance to **get health services** so it was costful also we losted our beloved ones a mother or child because of delivering on the way to the health facility and some women decided to deliver at home because they couldn't afford the cost to go to the health center all these led to maternal deaths in our community but now situation has been improved (TZ1_L2)	- Ceux qui souffrent là risque de mourir dans leur maisons car ils n'ont plus **rien a mangé** (BF1_L2)<br>- Depuis que la mine est arrivée vraiment il n'y a plus la santé et les gens **ne vivent plus longtemps** (BF3_L1)<br>- Tem muitas doenças que **nós mata** (MZ2.2_L2)<br>- Aqueles que têm coragem vão lá mesmo no mato, mesmo que **possam ser mortos**, para tentar cava (MZ3_L7)<br>- When the houses have cracks the vibrations may cause the house to fall down and **you will all di**e in there (TZ3_L3)<br>- Going to steal remaining of the processed stones called "magwangala", so **the security guard kills them** for self-defence (TZ3_L1)<br>- The use of unsafe water is what causes **miscarriage** (TZ2_L2)<br>- If you consider all these factors the answer is people from [our village] are **the living dead** (they are expecting to die) because they are using things which are not safe (TZ3_L7)
**Well-being**		
	- Les gens arrivent à **entretenir** leurs enfants par rapport à avant, car ils y'a des parents qui travaillent à la mine (BF3_L5)<br>- Drilled well has improved **healthy** because water is safe (TZ1_L6)<br>- The ENREP [natural resource extraction project] has constructed school, classrooms and toilets which has improved our **well-being** (TZ2_L5)<br>- So when mining built this health center here we **feel good**, we get treatment near our homes and we don't waste transport fee as we used to do before (TZ2_L7)	- Nous n'avons **plus une bonne qualité de vie**, c'est pas facile de vivre permanant «en bas de la poussière» (BF2_L8)<br>- Il n'y a pas d'eau, pas de travail, c'est la **misère** (BF1_L2)<br>- Quand ils font le dynamitage, on sursaute de **peur** à chaque moment (BF1_L4)<br>- Comemos mal, **dormimos a maneira**, as nossas machambas já reduziam até 50 metros e o resto são desses brancos (MZ2.2_L3)<br>- People are currently living with **fear** because boundaries between the community and mining are not clear (TZ1_L5)<br>- We **do not trust** the water that we use (TZ1_L5)<br>- We are in a very **difficult situation** because the poisons they using are dangerous (TZ3_L4)<br>- We have been **weak** due to lack of money from mining jobs (TZ2_L2)
