# Supplementary material for: Health impacts of industrial mining on surrounding communities: Local perspectives from three sub-Saharan African countries
Source: PLoS One. 2021 Jun 4;16(6):e0252433. doi: 10.1371/journal.pone.0252433 (PMC8177516; doi:10.1371/journal.pone.0252433)
Supplement: Attachment — Submitted filename: PLOS REVIEW.docx [file pone.0252433.s005.docx]

Greetings,

I would like to acknowledge the authors in conducting this study. It is a very challenging research area and involves a huge planning and co-ordination which is a laborious task.

Overall the study provides an in-depth insight to the health problems and other factors determining the health among the communities around the industrial mining sites.

**Abstract :** Appropriately covers the content and well presented.

**Main Article:**

Introduction: Very elaborate and covers the required information with lacunae in existing literature also justifies the need

**Methodology:** The methodology could be added with more details on how the sample size was achieved? Whether the study subjects included even those who were employed in the mining industries? If so whether there was any bias in the elicited impacts? How is it taken care?

**Results:** Appropriate

**Discussion:** Elaborate and appropriate

Conclusion: The details on key determinants impacted could be included along with this sentence: ‘Community perspectives revealed various impacts on the wider determinants of health and health outcomes related to morbidity, mortality and well-being’
